# Supplementary material for: The metabolically protective energy expenditure increase of Pik3r1-related insulin resistance is not explained by Ucp1-mediated thermogenesis
Source: Am J Physiol Endocrinol Metab. Author manuscript; Available in PMC 2025 Jul 5. (PMC7617857; doi:10.1152/ajpendo.00449.2024)
Supplement: Supplemental Material [file EMS206262-supplement-Supplemental_Material.zip › Luijten_et_al_Supplementary_Material.pdf]

Supplementary Material

The metabolically protective energy expenditure increase of *Pik3r1*-related insulin resistance is not explained by Ucp1-mediated thermogenesis

Ineke Luijten[1], Ami Onishi[1], Eleanor J. McKay[1], Tore Bengtsson[2], Robert K. Semple[1,3]

[1] Centre for Cardiovascular Science, University of Edinburgh, Edinburgh, UK

[2] Department of Molecular Biosciences, The Wenner-Gren Institute, Stockholm University, Stockholm, Sweden

[3] MRC Human Genetics Unit, Institute of Genetics and Cancer, University of Edinburgh, Edinburgh, UK

Am J Physiol Endocrinol Metab 2025

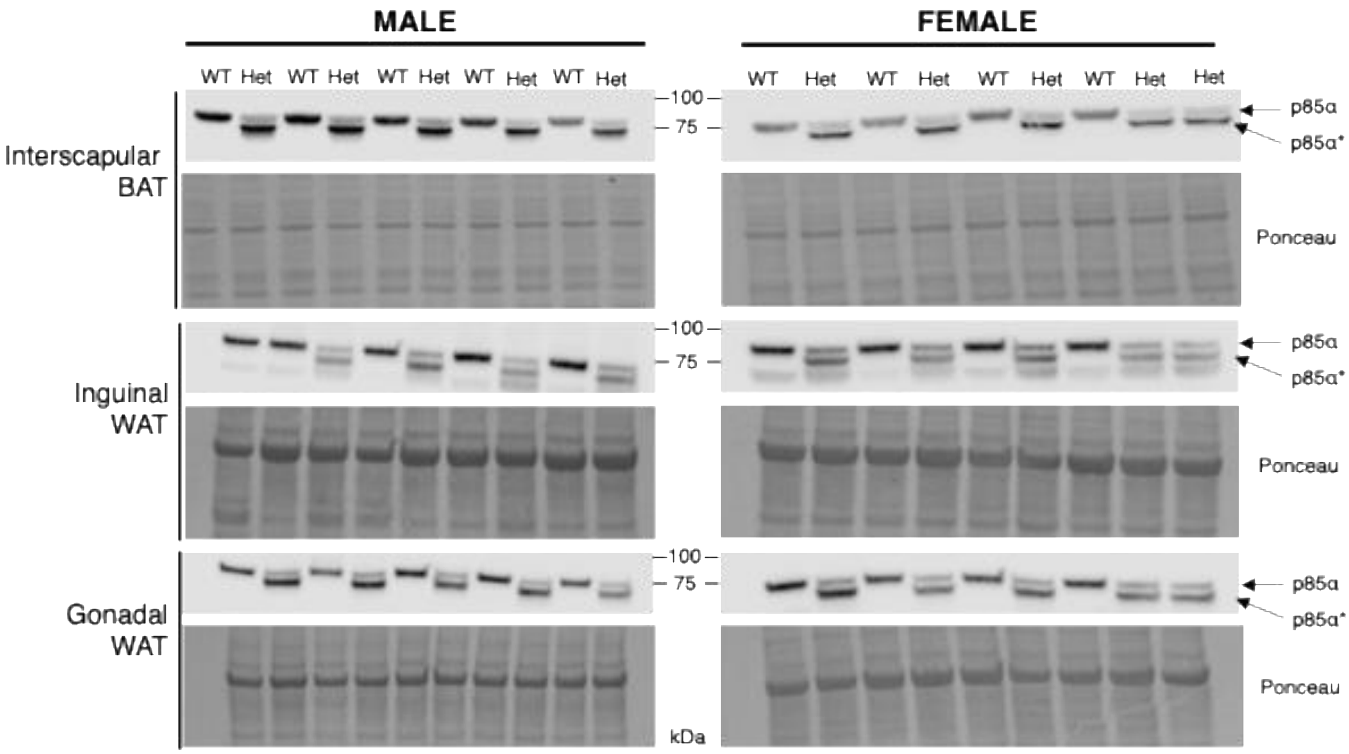

Supplementary Figure 1: Confirmation of Pik3r1 Y657X Expression in Adipose Tissue of *Pik3r1$^{Y657X/WT}$* mice . Immunoblots of 10 male (5 *Pik3r1* wild-type (WT) and 5 Pik3r1 Y657X heterozygous (Het)) and 9 female (4 WT; 5 Het) are shown. The truncated Pik3r1 Y657X is clearly visible in all heterozygous mice and is labelled as Pik3r1α*. Ponceau Red-stained membranes are shown as a loading control.

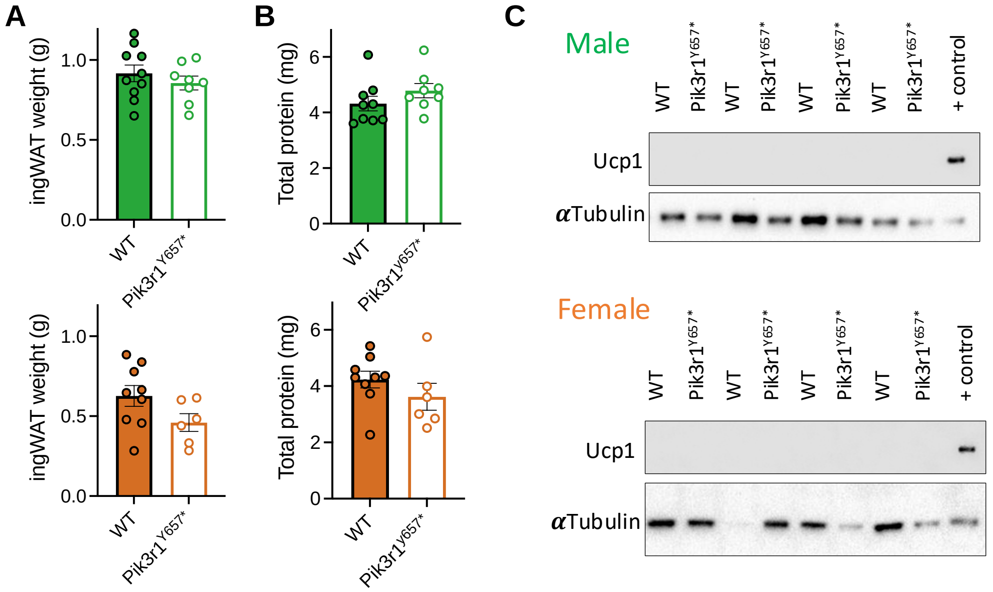

Supplementary Figure 2, Related to Fig. 3. Ucp1 protein amount in inguinal WAT at 21 °C. Male (green) and female (orange) WT and Pik3r1^{Y657*} mice were fed a 45% HFD and kept at 21 °C for the duration of the study. (A) Total ingWAT weight. (B) Total protein per depot. (C) Representative immunoblots showing Ucp1 protein in BAT homogenates with αTubulin as a loading control. All data are represented as mean ± SEM; Male WT N = 10, Male Pik3r1^{Y657*} N = 8, Female WT N = 9, Female Pik3r1^{Y657*} N = 6. Student's t test (all).

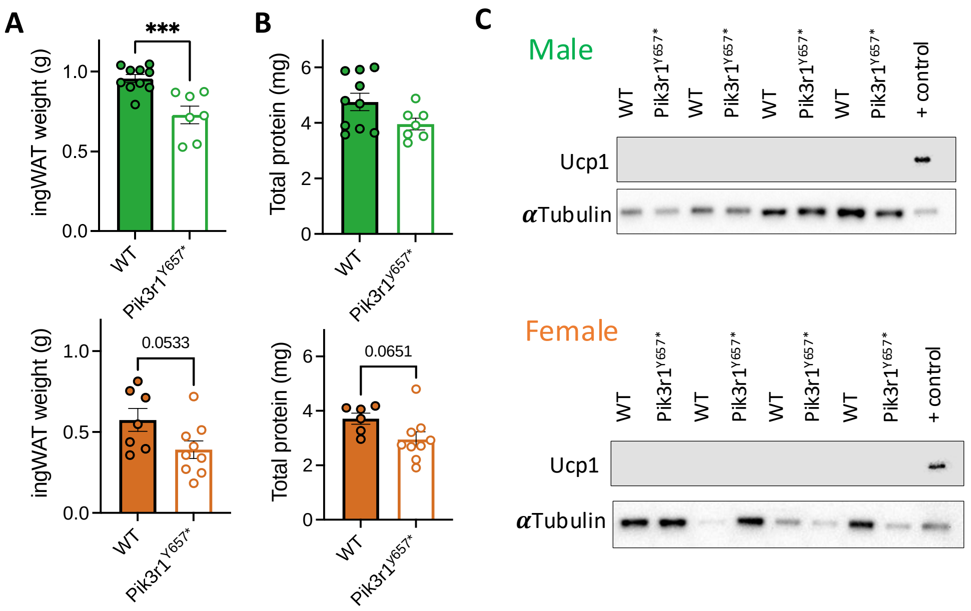

Supplementary Fig. 2, Related to Fig 5. Ucp1 protein amount in inguinal WAT at 30 °C. Male (green) and female (orange) WT and Pik3r1^{Y657*} mice were fed a 45% HFD and kept at 30 °C for the duration of the study. (A) Total ingWAT weight. (B) Total protein per depot. (C) Representative immunoblots showing Ucp1 protein in BAT homogenates with αTubulin as a loading control. All data are represented as mean ± SEM; Male WT N = 10, Male Pik3r1^{Y657*} N = 7, Female WT N = 7, Female Pik3r1^{Y657*} N = 9. *** p<0.001, Student's t test (all).
